# A Pre–Post Study of the Feasibility, Acceptability and Benefits of a Co‐Design Approach for the Development of a Digital Suicide Prevention App for International Students

**DOI:** 10.1111/hex.70669

**Published:** 2026-04-13

**Authors:** Christina Ng, Tanvi Shaikh, Jo Robinson, Jennifer Nicholas, Vivienne Browne, Gina Chinnery, Isabella Choi, Bailey Nation‐Ingle, Kristal Allison, Ella Perlow, Michelle Lamblin, Ellie Brown, Gregory Armstrong, Madhavan Mani, Samuel McKay

**Affiliations:** ^1^ Orygen Parkville Victoria Australia; ^2^ Centre for Youth Mental Health The University of Melbourne Melbourne Victoria Australia; ^3^ Sydney Medical School, Faculty of Medicine and Health The University of Sydney Sydney New South Wales Australia; ^4^ Suicide Prevention and Response Office, Victorian Department of Health Victorian Government Melbourne Victoria Australia; ^5^ The Australian Research Council (ARC) Centre of Excellence for Children and Families Over the Life Course (the Life Course Centre) Parkville Victoria Australia; ^6^ Centre for Mental Health and Community Wellbeing, Melbourne School of Population and Global Health The University of Melbourne Melbourne Victoria Australia; ^7^ Thriver Consulting Moggill Queensland Australia

**Keywords:** co‐design, culturally and linguistically diverse (CALD), digital intervention, international students, participatory research, PPI, suicide prevention

## Abstract

**Background:**

Interest in co‐design for suicide prevention is growing, yet few studies have evaluated feasibility, acceptability and impact, particularly for culturally and linguistically diverse populations. This study assessed a co‐design process used to develop a digital suicide prevention intervention with international students in Australia.

**Design:**

We conducted a mixed‐methods pre–post evaluation across 10 student and three stakeholder workshops. Participants were 27 international students (57.7% with lived experience of suicidal thoughts and behaviours) from 18 countries and 21 sector stakeholders (education, well‐being and student services). Surveys assessed feasibility, acceptability, safety, satisfaction, perceived benefits and confidence in co‐design skills. Quantitative data were analysed using descriptive statistics and paired *t*‐tests; open‐ended responses were thematically summarised.

**Results:**

Workshops were feasible, with high ratings for accessibility, support and safety. International students reported high acceptability and satisfaction, with significant increases in co‐design confidence (e.g., collaborating, adapting, participating and influencing outcomes) and perceived benefits (greater mental‐health literacy, empowerment, social connection and belonging). Stakeholders reported moderate to high acceptability and valued cross‐sector learning and system insights; confidence ratings showed no measurable change. Safety ratings were high for both groups.

**Conclusions:**

Co‐design was feasible, acceptable and safe, and associated with benefits for participants. Findings support co‐design as a practical, capacity‐building approach for developing culturally responsive digital suicide‐prevention initiatives for culturally and linguistically diverse populations.

**Patient or Public Contribution:**

An expert advisory group of international students with lived experience of suicide provided ongoing guidance. International students and sector stakeholders co‐designed the intervention and contributed to planning, design, implementation and review.

## 1 Introduction

Traditional, top‐down approaches to mental health program development often fail to meet the needs of diverse populations [1]. In suicide prevention, co‐design, the collaborative creation of prevention initiatives, interventions or resources with end users, is increasingly used as a means to enhance cultural relevance, feasibility and effectiveness by involving individuals with lived experience in shaping interventions [2, 3]. Despite this, empirical research on co‐design in suicide prevention remains limited [4, 5]. Most studies provide descriptive accounts or focus group summaries, with little evaluation of outcomes such as the acceptability and safety of engaging in co‐design processes on this topic, or the participant‐perceived benefits of the process [6, 7]. Few report how power was shared or how input shaped final designs, raising concerns about the depth of engagement [8].

These gaps in understanding are potentially wider when engaging with culturally and linguistically diverse (CALD) populations, who face unique barriers to mental health care, including stigma, language difficulties and a lack of culturally appropriate services [9]. Although co‐design is often presented as culturally inclusive, little research has examined how CALD participants experience and influence these processes [10]. Juras et al. highlight the need to explore how power and representation are negotiated across cultural contexts and how these dynamics affect the relevance and impact of co‐designed interventions [10]. Key questions remain about the conditions required for safe, meaningful participation and the benefits of co‐designing with these communities.

International students in tertiary education (e.g., post‐secondary vocational education and training or university) settings represent a CALD population that is both high‐risk and underserved in suicide prevention. International students report elevated rates of suicidal thoughts and behaviours [11], yet have few interventions tailored to their cultural and structural contexts [12]. Studies across host countries show that international students experience a unique combination of risk factors for suicidal thoughts and behaviours related to living and studying in another country, while also negotiating existing cultural views of mental health and suicide [11, 13, 14]. Previously identified risk factors include a lack of belonging [15], experiences of discrimination [16], unmet academic performance expectations [17], perceived public stigma [18] and low mental‐health literacy [19]. This population is also highly diverse in nationality, language and belief systems, making them an important case study for how co‐design can produce culturally responsive and scalable suicide‐prevention initiatives within diverse groups [20].

Digital interventions (e.g., activities accessed via technology platforms such as computers, smartphones and virtual reality) that can improve users' mental health are increasingly used in suicide‐prevention initiatives because they are accessible, scalable and adaptable to diverse users [21, 22, 23]. Self‐guided, low‐intensity digital interventions have demonstrated efficacy in reducing self‐harm and suicidal ideation [24] and may be particularly well‐suited for international students who face cultural and structural barriers to traditional support, including limited English proficiency, financial constraints and unfamiliarity with local health systems [20]. However, it is increasingly recognised that these interventions only work when they reflect the specific cultural contexts, norms and day‐to‐day experiences of the people who use them.

### 1.1 Current Study

In the current study, we aimed to evaluate participants' experiences of co‐designing a suicide prevention app for international students from concept development to dissemination. We employed a quantitative pre‐post design to assess changes in suicide prevention knowledge, confidence and readiness to engage in co‐design, responding to calls for more rigorous evidence in this area [6, 8]. We collaborated with both international students and sector stakeholders to ensure the intervention was grounded in lived experiences and system realities, as such approaches can enhance the cultural relevance, feasibility and sustainability of mental health initiatives [1, 2]. The study addressed the following research questions (RQs):

1. Is co‐design feasible to deliver with international students and sector stakeholders in tertiary education settings?
2. Do participants find the co‐design workshops acceptable, including satisfaction with processes and activities?
3. Are the workshops experienced as psychologically and culturally safe?
4. What benefits, if any, do participants gain from taking part in co‐design workshops?

## 2 Methods

Ethical approval for this study was obtained from the University of Melbourne Human Research Ethics Committee (reference number: 30125), and the study complied with the Declaration of Helsinki. Patient and public involvement processes are reported in accordance with the GRIPP2 long‐form checklist [25] (see Supporting Information: Appendix 1).

### 2.1 Study Design

A pre–post design assessed the impact of co‐design workshops. Participants completed online surveys before (T1) and immediately after (T2) the workshop. Before attending the workshops, participants provided informed consent and completed a wellness plan (students only). Participants who attended additional workshops just completed a short follow‐up survey to reduce response burden. Up to three reminder emails were sent to maximise follow‐up. International students received $90 AUD per workshop.

### 2.2 Participants

#### 2.2.1 International Students

Recruitment occurred via snowball sampling through institutional contacts and student networks, with Expressions of Interest (EOIs) managed in REDCap (*n* = 856). Eligible participants were international students ≥ 18 years enrolled at tertiary institutions in Victoria. Stratified sampling by institution, nationality, age and study major was used to obtain a representative sample. The final sample (*n* = 27) was predominantly female (66.6%), with a mean age of 26.4 years (SD = 6.1), and enrolled mainly at the University of Melbourne (66.7%). Students represented Asia, Africa, Europe and Latin America; the largest groups were Vietnamese (14.8%) and Chinese (11.1%). Over half reported lifetime lived experience of suicidal thoughts (57.7%) or self‐harm (50.0%), and 15.4% reported a suicide attempt (see Supporting Information: Table 1 for a full breakdown). Students' goals for the workshops included advocating for greater support (85.2%), contributing to suicide prevention research (74.1%) and learning about suicide prevention (66.7%; see Supporting Information: Table 2 for a full summary).

#### 2.2.2 Sector Stakeholders

Stakeholders were recruited through snowball sampling using the research team's professional networks across the international education and student support sectors. Twenty‐nine stakeholders registered, and 21 attended workshops. Most were from universities (57.1%) or TAFEs (Technical and Further Education; 23.8%), with roles spanning counselling/mental health (52.2%), student affairs (30.4%) and orientation/transition (30.4%; see Supporting Information: Table 3). Participants averaged 6.7 years' experience (SD = 6.4) and 28.6% identified as belonging to a marginalised group (e.g., LGBTQIA+, disability, culturally or linguistically diverse). Their goals for the workshop were to contribute to suicide prevention research (81.0%), collaborate with peers (76.2%) and explore online tools (76.2%; see Supporting Information: Table 2).

### 2.3 Co‐Design Workshops

Thirteen workshops were held: 10 with students and three with stakeholders. Participants were required to attend at least one workshop; subsequent attendance was optional. Planning drew on Bowen et al.'s five‐stage model, which provides a structured, iterative process for participatory intervention development comprising (1) understanding experiences, (2) exploring ideas, (3) selecting concepts, (4) converging on practical proposals and (5) prototyping and evaluating [26]. This model was selected because it provides a structured, iterative process for stakeholder engagement and refinement, enabling progression from lived experience insights to feasible, culturally responsive intervention components. Workshop design was also guided by best practice guidelines for engaging CALD communities [27] and young people with lived experience of suicide in participatory research [28, 29]. See Supporting Information: Appendix 2 for a summary of the workshops, including all activities and outcomes.

A youth expert advisory group of five international students with lived experience, recruited via existing research participant networks and Study Melbourne (international student support service) social media channels, met monthly and provided ongoing input into planning and facilitation, receiving 90 AUD per month in remuneration for their time. Before commencing, students were onboarded through the institution's standard onboarding process for youth participants, including completing a police check, a Working with Children check and a participation agreement that outlines roles, rights and responsibilities. They also completed wellness plans that covered mental health issues and preferred supports.

To promote equitable collaboration, members co‐defined ground rules, meeting formats and preferred input methods (e.g., group discussion, written feedback) with researchers. Meetings were offered in person and online; after the first session, members elected to continue online. The advisory group provided input on workshop plans, content and support materials, and reviewed intervention concepts and prototypes throughout the workshop cycle. Members were also invited to attend workshops as co‐facilitators (an additional 90 AUD per workshop was provided) and to contribute to manuscript development, with one member (T.S.) choosing this option. Participation also provided opportunities to develop co‐design and facilitation skills, contribute to suicide‐prevention initiatives and gain professional experience.

The facilitation approach prioritised psychological safety, reciprocity and ensuring that participants could clearly see their ideas shaping the workshop's direction. Sessions began with grounding or icebreaker activities and used scaffolded creative tasks with plain‐language prompts and visual tools (e.g., journey maps, card sorts) to support equal participation across language backgrounds. Facilitators modelled openness and curiosity, validated lived‐experience insights and linked participant suggestions to design decisions in real time. Visual voting boards made these linkages explicit, reinforcing transparency, trust and collective agency. These methods fostered an environment in which students felt safe contributing ideas and experienced genuine influence over the intervention's direction (see Supporting Information: Appendix 3 for an overview of the co‐designed intervention).

#### 2.3.1 International Student Workshops

Each workshop was co‐facilitated by a peer researcher, a member of the youth advisory group and a researcher with experience in education‐based mental health initiatives, all of whom had lived experience as international students. Workshops lasted 3 h, followed a consistent structure (icebreaker, co‐design activities, review and cooldown, and post‐workshop survey) and were supported by an on‐call psychologist. To enhance accessibility and inclusiveness, sessions incorporated low‐level background music, fidget toys, live AI‐based transcription for language support and anonymous digital feedback tools (e.g., Mentimeter) to enable written participation. Participants received printed handouts created in consultation with the advisory group that covered helpful definitions, suicide‐safe language and co‐design principles to support a shared understanding of key terms and concepts (see Supporting Information: Appendix 4 for these resources). Eight sessions were delivered in person and two online (Microsoft Teams with Miro). Students attended on average 3.3 workshops (SD = 1.8, range = 1–7). Workshops averaged nine attendees (SD = 1.8, range = 7–12), with 23 students attending multiple sessions, yielding 90 total attendances across 27 students. Survey completion was high (*n* = 26, 96.3% pre; *n* = 73, 81.1% post).

#### 2.3.2 Sector Stakeholder Workshops

Stakeholder workshops were conducted online (using Microsoft Teams and Miro) and lasted 2 h, following the same structure as the student workshops. A peer researcher and an experienced mental health researcher facilitated them. Participants provided informed consent and completed pre‐workshop surveys. Support was provided via helplines and access to an on‐call psychologist outside of sessions. Stakeholders were not financially compensated, as participation occurred during working hours and attended on average 1.2 workshops (SD = 0.4, range = 1–2), with four attending multiple sessions. Workshops averaged 8.3 attendees (SD = 2.5, range = 6–11), yielding 25 total attendances across 18 individuals. Survey completion was high pre‐workshop (*n* = 20, 95.2%) but lower post‐workshop (*n* = 17 68.0%).

### 2.4 Measures

There are currently no widely validated measures for assessing co‐design experiences and outcomes. Recent work in the #chatsafe co‐design program (La Sala et al., unpublished) [30] developed questions to assess feasibility, acceptability, satisfaction and safety. These questions were based on the Youth Engagement Evaluation Framework [31], the Theoretical Framework of Acceptability [32] and prior participatory suicide prevention studies [4, 33]. However, these #chatsafe‐origin questions did not capture experiences specific to CALD participants or outcomes related to co‐design skill development. Accordingly, we adapted several of their questions and developed new items where none were available, guided by the same theoretical frameworks (see Supporting Information: Appendix 5 for the full measures). Surveys were administered via REDCap.

#### 2.4.1 Demographic Information

International students indicated their age, gender, sexuality, nationality, cultural/ethnic identity, religious beliefs, duration of residence in Australia, languages spoken, education, employment and lived/living experience of suicide and self‐harm. Sector stakeholders reported their role, organisation type, experience in the education sector and minority group identification.

#### 2.4.2 Feasibility

Feasibility was assessed at T2 using four questions developed for the #chatsafe co‐design program (La Sala et al., unpublished) [30] rated on a 5‐point Likert scale (1 = *Strongly disagree*, 5 = *Strongly agree*). Questions included perceptions of ease of participation, convenience, support and fair compensation.

#### 2.4.3 Acceptability

At T1, participants completed 12 questions developed for the #chatsafe co‐design program (La Sala et al., unpublished) [30] assessing motivations and expectations for the workshop. At T2, an extended version of these #chatsafe‐origin questions assessed inclusion, usefulness, social connection and diversity of voices, with additional items capturing power distribution, support, cultural responsiveness and access to resources, all rated on a 5‐point Likert scale (1 = *Strongly disagree*, 5 = *Strongly agree*). Another T2 item (first attendance only) asked whether the workshop met expectations.

#### 2.4.4 Satisfaction

Satisfaction at T2 was assessed using (1) a single‐item question for international students on overall satisfaction with the workshop and (2) an 8‐item set derived from questions developed for the #chatsafe co‐design program (La Sala et al., unpublished) [30] administered at first attendance only. The original #chatsafe wording (e.g., ‘Creating social media content’, ‘Learning about #chatsafe’) was modified to reflect the present co‐design process (e.g., ‘Working on the new intervention’, ‘Learning about suicide prevention’).

#### 2.4.5 Safety

At T2, five questions developed for the #chatsafe co‐design program (La Sala et al., unpublished) [30] were used to assess emotional and psychological safety, cultural safety and perceived risk of harm on a 5‐point Likert scale (1 = *Strongly disagree*, 5 = *Strongly agree*). The only modification was changing references from ‘#chatsafe co‐design workshop’ to ‘co‐design workshop’.

#### 2.4.6 Confidence in Co‐Design Skills

Perceptions of confidence in their co‐design skills was assessed at T1 and T2 (first attendance only) using 11 items rated on a 5‐point Likert scale (1 = *Not at all confident*, 5 = *Very confident*) measuring participants' confidence in understanding workshop goals, contributing, collaborating, receiving and incorporating feedback, adapting, participating in activities, impacting outcomes, managing power differences, caring for their well‐being, seeking support and sharing lived experiences.

#### 2.4.7 Benefits

Perceived benefits from the workshops were assessed at T2 (first attendance only) using a 13‐item scale covering knowledge of mental health, suicide prevention and support, as well as feelings of empowerment and value, social connection, belonging and help‐seeking intentions. In addition, after each workshop, participants responded to a separate open‐ended question about what they found most valuable from the workshop.

#### 2.4.8 General Feedback Question

All participants were invited to provide open‐ended feedback about their workshop and survey experience.

### 2.5 Analytic Approach

The data were analysed using RStudio. Summary statistics and scores for each measure were calculated, and paired samples *t*‐tests were used to examine the changes in perceived confidence in co‐design skills from pre‐ to post‐workshop among participants attending their first workshop. Analyses used complete‐case data; participants with missing T2 responses for a given question were excluded from the paired *t*‐test for that question. No imputation was performed. The first author mapped open‐text responses to the RQs, with illustrative quotations selected to contextualise and elaborate the quantitative findings.

### 2.6 Use of Artificial Intelligence Tools

ChatGPT (OpenAI, GPT‐5.1) was used only for text editing and wording refinement. All content was reviewed and verified by the authors. No analyses, results or interpretations were generated by the tool.

## 3 Results

### 3.1 RQ 1: Feasibility

#### 3.1.1 International Students

Feasibility ratings were uniformly high: 100% (agreed/strongly agreed) found participation easy, convenient and fairly compensated, and 98.6% felt supported. High interest in co‐design workshops with 856 EOIs for participation, and most (85.2%) participants attending multiple workshops further support the feasibility. A summary of the feasibility ratings for international students and sector stakeholders is shown in Table 1.

**Table 1:** Detailed workshop acceptability for first‐time attendees.

Item	Students	Stakeholders
Easy to participate	4.77 (0.43)	3.93 (1.00)
Felt supported	4.73 (0.53)	4.43 (0.65)
Convenient to participate	4.69 (0.47)	4.00 (0.96)
Fair payment	4.58 (0.50)	3.21 (1.31)

*Note:* Values in parentheses represent standard deviations (SD). Student *n* = 27, stakeholder *n* = 14. Stakeholder sessions were held during working hours with no honorarium.

#### 3.1.2 Sector Stakeholders

Perceived feasibility was high for support (92.9% agree/strongly agree) and acceptable for ease (78.6%) and convenience (71.4%), with small minorities disagreeing (none strongly on ease). Fair compensation drew mixed ratings (35.7% agree; 42.9% neutral; 21.4% disagree, incl. 14.3% strongly). Engagement was moderate (EOIs = 29) with lower repeat attendance compared to the student workshops.

### 3.2 RQ 2: Acceptability and Satisfaction

#### 3.2.1 Acceptability

##### 3.2.1.1 International Students

Acceptability was very high across all 12 items. Full endorsement (100% agreed/strongly agreed) was observed for feeling included, finding participation useful, fair distribution of power, equal opportunities to share and respect for cultural backgrounds. High agreement (> 95%) was reported for feeling supported, socially connected and having needs met, with the lowest rating (92.6%) for having all necessary resources. Most participants reported that their goals were met (88.8%), although 11.1% strongly disagreed, suggesting a small subgroup whose expectations differed. In addition, students (96.3%) reported very high overall enjoyment of the workshops. A summary of the acceptability ratings for international students and sector stakeholders is shown in Table 2.

**Table 2:** Detailed workshop acceptability for first‐time attendees.

Item	Students	Stakeholders
I felt included in the co‐design process	4.78 (0.42)	4.07 (0.92)
My needs were met in the co‐design process	4.67 (0.55)	3.79 (1.05)
Participating in the co‐design workshop was useful	4.67 (0.48)	3.86 (1.23)
I felt I connected socially and meaningfully with other participants in this workshop	4.59 (0.57)	4.14 (1.03)
A diverse range of voices and experiences were included in the workshop	4.67 (0.48)	3.64 (1.22)
The workshop was representative of my opinions	4.70 (0.47)	3.79 (0.97)
Power was distributed fairly between participants and researchers	4.78 (0.42)	4.14 (0.86)
All participants had equal opportunities to share their ideas and perspectives	4.81 (0.40)	4.07 (0.73)
The facilitators created a supportive and emotionally safe environment	4.81 (0.48)	4.21 (0.80)
The cultural backgrounds and values of all participants were respected and included	4.85 (0.36)	4.36 (0.74)
I felt that my expertise was valued and utilised in the workshop	4.59 (0.57)	4.29 (0.73)
I had access to all the resources and information I needed to participate fully	4.63 (0.63)	4.14 (0.77)
Degree enjoyed workshop	4.74 (0.53)	4.00 (0.96)
Workshop met expectations and goals	4.15 (1.23)	3.71 (1.27)

*Note:* Values in parentheses represent standard deviations (SD). Student *n* = 27 and stakeholder *n* = 14.

##### 3.2.1.2 Sector Stakeholders

Stakeholders rated the workshops positively but with more variability. The strongest endorsements were for cultural respect (85.7%), feeling valued (85.7%) and fair power distribution (85.7%). Majorities also agreed that facilitators created a safe environment (78.6%), that they felt included (78.6%) and that they had access to needed resources (78.6%). Areas with lower endorsement included diversity of voices (71.4%) and usefulness (71.4%), and the lowest was needs being met (64.3%). Notably, there was no disagreement for several core items (cultural respect, safety and equal sharing opportunities), highlighting broad acceptance even where improvement was possible.

#### 3.2.2 Satisfaction

##### 3.2.2.1 International Students

Students reported very high satisfaction (93.2%), with universal endorsement for facilitators and collaboration opportunities, and strong ratings for design activities, social connection and available resources (> 90%). A summary of the satisfaction ratings for international students and sector stakeholders is shown in Table 3.

**Table 3:** Workshop participant satisfaction ratings.

Item	Students	Stakeholders
Overall satisfaction	4.52 (0.97)^a^	Not reported
Facilitators	4.85 (0.36)	4.50 (0.76)
Design activities	4.59 (0.57)	4.07 (1.21)
Connecting socially with others	4.56 (0.58)	3.43 (1.28)
Working collaboratively	4.78 (0.42)	3.71 (1.14)
Connecting with professionals	4.48 (0.70)	3.64 (1.22)
Working on intervention	4.44 (0.75)	3.93 (1.27)
Learning about suicide prevention	4.37 (0.79)	3.50 (1.51)
Resources available	4.44 (0.64)	3.79 (1.31)

*Note:* Values in parentheses represent standard deviations. Student *n* = 27, stakeholder *n* = 14.

^a^ *n* = 73.

##### 3.2.2.2 Sector Stakeholders

Satisfaction was highest for the facilitators (85.7% agreed/strongly agreed), followed by working on the intervention (71.4%) and the design activities (64.3%). Moderate endorsement was reported for resources available (57.1%), working collaboratively (50.0%) and connecting with professionals (50.0%). Lower agreement was observed for connecting socially with others (42.9%) and learning about suicide prevention (42.9%). Notably, there was no disagreement on facilitators (0% rated 1–2), whereas small pockets of disagreement appeared for social connection and learning (both 21.4%). Overall, stakeholders rated facilitators very positively, with mixed views on social connection and learning.

### 3.3 RQ3: Safety

#### 3.3.1 International Students

Students overwhelmingly reported that workshops were psychologically and culturally safe. Most disagreed that participation made them want to self‐harm (94.5%), feel upset (91.8%) or suicidal (94.5%). Strong endorsement was observed for feeling psychologically (91.8%) and culturally (95.9%) safe. A small subgroup (5.5%) strongly agreed with all items (including distress items). In comparison, another subgroup (2.7%) strongly disagreed with all items (including safety items), likely indicating straight‐line response patterns rather than genuine harm. Follow‐up confirmed no participants were in acute distress as a result of the workshops. A summary of the safety ratings for international students and sector stakeholders is shown in Table 4.

**Table 4:** Workshop safety ratings.

Item	Students	Stakeholders
I felt upset during the workshop	1.38 (1.01)	1.35 (1.00)
I felt suicidal during the workshop	1.26 (0.93)	1.24 (0.97)
I felt like self‐harming during the workshop	1.27 (0.93)	1.24 (0.97)
I felt emotionally/psychologically safe	4.58 (0.98)	4.65 (0.70)
I felt culturally safe	4.63 (0.77)	4.65 (0.70)

*Note:* Values in parentheses represent standard deviations. Student *n* = 73, stakeholder *n* = 17.

#### 3.3.2 Sector Stakeholders

Stakeholder findings were similar with 94.1% disagreeing with distress items and 88.2% endorsing psychological and cultural safety, and no participants reporting harm. One stakeholder also displayed a straight‐line response pattern with top scores for both distress and safety.

### 3.4 RQ 4: Benefits

#### 3.4.1 Confidence in Co‐Design Skills

##### 3.4.1.1 International Students

Students showed significant pre–post gains across most items after attending their first workshop, including understanding workshop goals, collaborating, incorporating feedback, adapting to change, participating in activities, influencing outcomes and handling power differences (all *p* < 0.05). Smaller, non‐significant gains were seen in self‐care, seeking support and sharing lived experiences safely (see Table 5 for a summary for students and stakeholders).

**Table 5:** Confidence scores for co‐design skills pre‐ and post‐workshop for first‐time attendees.

Item	International students				Stakeholders			
*Confidence to do the following:*	T1	T2	*T*	*P*	T1	T2	*T*	*P*
Understand workshop goals	4.27 (0.83)	4.77 (0.43)	3.35	0.003**	4.29 (0.47)	4.21 (0.80)	0.25	0.807
Meaningfully contribute	4.31 (0.79)	4.54 (0.65)	1.36	0.185	4.14 (0.66)	4.00 (0.96)	0.52	0.612
Work collaboratively	4.27 (0.78)	4.69 (0.47)	2.85	0.009**	4.57 (0.51)	4.14 (0.86)	1.47	0.165
Receive and incorporate feedback	4.27 (0.78)	4.65 (0.49)	3.08	0.005**	4.50 (0.65)	4.00 (0.88)	1.71	0.110
Adapt to changes	4.31 (0.68)	4.65 (0.49)	2.81	0.010*	4.36 (0.50)	4.00 (0.96)	1.05	0.315
Participate in co‐design activities	4.23 (0.86)	4.73 (0.45)	2.82	0.009**	4.36 (0.50)	4.00 (0.78)	1.44	0.174
Impact designs/project outcomes	3.85 (0.92)	4.54 (0.58)	3.80	0.001**	3.93 (0.62)	3.86 (0.95)	0.27	0.793
Handle power differences	4.19 (0.75)	4.50 (0.65)	2.13	0.043*	4.00 (0.68)	3.79 (0.58)	1.00	0.336
Care for well‐being	4.38 (0.80)	4.50 (0.58)	0.83	0.416	4.36 (0.50)	4.07 (0.62)	1.30	0.218
Seek support	4.35 (0.75)	4.42 (0.64)	0.53	0.603	4.43 (0.65)	4.07 (0.73)	1.79	0.096
Share lived experiences safely	4.15 (0.83)	4.50 (0.58)	1.89	0.071	4.29 (0.83)	3.93 (0.73)	1.59	0.136

*Note:* Values in parentheses represent standard deviations (SD). *p < 0.05, **p < 0.01, ***p < 0.001, student *n* = 26, stakeholder *n* = 14.

Qualitative reflections echoed these findings, where many described increased confidence in expressing their ideas. One participant noted they felt more confident in ‘expressing myself and being confident in my ideas’, while another shared they had learned to ‘speak without fear of judgment’. Others reported gains in collaborative problem‐solving, creative thinking and facilitation skills, with several highlighting the value of ‘listening’, ‘brainstorming under time pressure’ and ‘working with others from diverse backgrounds’. These reflections suggest that, beyond skill acquisition, students experienced meaningful growth, empowerment and peer connection through their engagement in the co‐design process.

##### 3.4.1.2 Sector Stakeholders

There were no statistically significant changes in sector stakeholders' confidence across the 11 assessed skill areas following the co‐design workshop, although most scores showed small non‐significant decreases. In contrast, qualitative feedback highlighted several areas of perceived skill development. Stakeholders reported improvements in their understanding of co‐design processes, confidence in using collaborative tools like Miro and their ability to summarise and communicate ideas. For example, one participant shared, ‘Being able to pull together and summarise ideas from others’, while another noted, ‘Learning about the use of Miro’. Others reflected on the collaborative aspect, stating they gained confidence in ‘working with others’ and ‘listening and sharing ideas with confidence’. These reflections suggest that the workshop provided an opportunity for stakeholders to consolidate their existing skills and engage in meaningful peer learning within a collaborative design environment.

#### 3.4.2 Perceived Benefits

International students reported strong perceived gains across knowledge, empowerment and connection domains. All felt their experience as an international student was valued (100%), and nearly all reported feeling empowered to improve their own mental health (96.3%) and support others (96.3%). High agreement (> 90%) was reported for increased research knowledge, greater social connection, greater likelihood of talking to friends about mental health and greater intention to contribute to prevention initiatives. Gains were slightly lower for the likelihood of seeking professional help if needed (81.5%; see Table 6 for more details).

**Table 6:** Students perceived benefits after their first co‐design workshop.

Item	*M* (SD)
I know more about research	4.44 (0.58)
I know more about suicide prevention	4.15 (0.77)
I know more about mental health	4.33 (0.83)
I am more aware of available mental health support services	4.26 (0.81)
I feel that I can contribute to suicide‐prevention initiatives	4.56 (0.64)
I feel empowered to improve my own mental health	4.56 (0.58)
I feel empowered to support others to improve their mental health	4.52 (0.58)
I feel a greater sense of belonging	4.30 (0.72)
I feel more socially connected	4.37 (0.63)
I feel like my experience as an international student is valued	4.63 (0.49)
I am more likely to seek professional help if experiencing mental health problems	4.37 (0.79)
I am more likely to talk to my friends about their mental health	4.44 (0.75)
I am more likely to recommend my friends seek professional help for their mental health problems	4.37 (0.74)

*Note: n* = 27.

#### 3.4.3 Value of the Workshops

##### 3.4.3.1 International Students

Participants consistently described the most valuable aspects of the co‐design process as sharing lived experiences, connecting with others and contributing meaningfully to the design of mental health resources. For first‐time attendees, themes encompassing shared experiences and connection, personal reflection and collaborative learning were most common. Many participants found comfort in hearing others' stories and realising they were not alone in their struggles: Knowing that I'm not alone in my struggles. Sometimes it really feels very isolating being an international student’. Activities also prompted personal insight: ‘Helped me reflect on the moment when I felt challenged and the journey of how I overcame it’. Others valued the collaborative aspects of the sessions: ‘Sharing ideas with a group’ and ‘I can learn different and creative ideas for mental health solutions in this workshop’.

Returning participants placed greater emphasis on hands‐on design, problem‐solving and witnessing the intervention's evolution: ‘Getting to design our own UI for the app’ and ‘Seeing how the new prototype has developed and incorporated our comments’. Collaboration and shared purpose remained central: ‘Everyone coming together with their own perspectives made me feel needed and heard’. These reflections highlight how co‐design empowered students not just as participants, but as co‐creators of solutions that reflect their lived realities.

##### 3.4.3.2 Sector Stakeholders

First‐time attendees appreciated the opportunity to share ideas, hear diverse perspectives and learn from others working in international student support. Discussions around service gaps and how the intervention could enhance support were seen as particularly valuable: ‘listening to how other institutions were experiencing and navigating the challenges… was validating’, while another appreciated ‘being in a group of like‐minded professionals who want to better this space’. Participants also noted the benefit of learning about the research and student contributions. While tools like Miro were useful for collaboration, some experienced technical challenges.

Returning stakeholders emphasised the value of continuity, collaboration and ongoing contribution. They appreciated the chance to deepen conversations and build on collective ideas: ‘the opportunity to share resources, experiences and connect with others’ and ‘providing our ideas and suggestions as people working with international students’. Overall, the workshops were seen as a meaningful space for cross‐sector connection and collaboration, reinforcing a shared commitment to improving international student well‐being.

## 4 Discussion

This study is the first to evaluate the feasibility, acceptability and benefits of co‐design in developing a digital suicide prevention intervention for international students in Australia. Overall, the workshops were feasible, acceptable and beneficial for both international students and sector stakeholders. Workshops were well‐attended and rated highly for accessibility and support, particularly among students. Stakeholders also rated feasibility positively but noted some areas for improvement, such as fairness of compensation and ease of participation. Safety was consistently rated high across sessions. Acceptability was strong among students, who reported high satisfaction with facilitation, collaboration and feeling included and respected. Stakeholders expressed moderate to high satisfaction and valued the facilitation, the opportunity to contribute and connect professionally with others, though some highlighted the need for greater representation and responsiveness to their needs. Lower ratings of fairness and repeat attendance were likely influenced by fewer available workshop slots and limited reimbursement.

Importantly, the workshops provided meaningful benefits for both groups. Students showed significant gains in confidence to engage in the co‐design process, especially in collaborative and reflective areas, and reported feeling more empowered, connected and informed about mental health. While stakeholders did not report significant quantitative gains, qualitative feedback highlighted professional learning, shared goals and cross‐sector dialogue as key outcomes. Students also described seeing their contributions directly shape the intervention over time. Collectively, these findings support the utility and value of co‐design as a participatory approach to developing culturally relevant, user‐informed mental health interventions.

These findings align with and build on existing literature highlighting the value of participatory design in youth and CALD communities, specifically in the method's ability to empower international students and support culturally sensitive adaptations of suicide prevention efforts [1, 2, 5]. A key outcome in prior research is that participatory approaches foster agency [4, 28, 33]. Similarly, students in our study reported gains in confidence, especially in collaborative and reflective skills, and in feeling heard, respected, and empowered in shaping a culturally relevant intervention. These outcomes reinforce the value of participatory methods for international student populations.

The strong focus on safety and comfort likely contributed to these positive outcomes and aligns with best practice guidelines [4, 5, 34]. Safety was embedded in each session through collaborative ground rules, wellness check‐ins, optional breaks and the presence of facilitators with diverse lived experiences. Calming elements such as background music and fidget items may also have reduced distress. Many participants had histories of self‐harm or suicidal ideation, yet safety scores remained high. Focusing workshops on designing solutions rather than eliciting personal narratives may have further mitigated emotional risk. These findings add to evidence that well‐facilitated co‐design can be both safe and effective in sensitive contexts [5, 8, 33, 35].

A key finding from this study is the powerful role of shared experiences among international students in shaping culturally responsive suicide prevention strategies. While the cultural diversity of international students is often seen as a barrier to designing effective interventions [12], our findings suggest it can be a strength. Students described how hearing diverse perspectives enriched the co‐design process, reduced feelings of isolation and deepened their understanding of mental health needs, findings similar to those of McKay et al. [5]. Together, these findings suggest that cultural diversity can be leveraged as a strength within co‐design, turning difference into a resource for innovation. Rather than designing for each cultural group separately, co‐design enabled students to surface shared challenges (e.g., isolation, academic pressure and navigating unfamiliar systems), learn from each other's coping strategies and collaborate on solutions that cut across cultural boundaries.

Another strength of this project was the sustained involvement of international students throughout all stages of co‐design. Unlike many past studies where youth or end users are only partially involved [1], this project engaged students throughout, from planning to prototype review. In addition to the participants, a dedicated youth advisory group informed workshop content, logistics and design decisions, ensuring the process remained grounded in student realities.

This inclusive approach also enhanced research engagement. Previous work highlights how meaningful co‐design can improve recruitment and data quality by fostering participant investment [1]. In our study, we observed exceptionally high interest among international students and moderate interest among sector stakeholders, reflecting the perceived relevance and resonance of the project. Survey completion rates were also high across pre‐ and post‐workshop time points. These patterns indicate the potential for participatory approaches to strengthen both intervention design and research engagement.

The inclusion of sector stakeholders added valuable perspectives and encouraged cross‐sector knowledge exchange [1, 2]. While no significant quantitative changes in stakeholder confidence were observed, qualitative feedback indicated greater understanding of systemic challenges and enhanced professional learning. Stakeholders valued the opportunity to share insights, reflect on service gaps and learn from how others approached similar issues. These findings suggest that participatory approaches can strengthen not only intervention relevance and student ownership but also shared responsibility and collaboration across education and mental health sectors.

## 5 Limitations

Several limitations should be noted. Differences in format likely contributed to variation in engagement and satisfaction between students and stakeholders. We also did not assess whether sector stakeholders viewed participation as a worthwhile use of their time, an outcome that may be important given competing workplace demands. Snowball sampling was used to recruit participants. All student participants and most stakeholders were based in Victoria, which may limit the generalisability to less engaged groups, other Australian jurisdictions and overseas contexts. Many participants did not report a history of suicide attempts, which limits generalisability to populations with higher attempt rates. Students attended primarily face‐to‐face sessions, which may have facilitated rapport and deeper interaction, whereas stakeholders joined online sessions, potentially limiting spontaneous discussion [36]. Digital literacy challenges may also have affected participation despite brief onboarding, suggesting that future workshops should use accessible platforms and provide live demonstrations [37]. To reduce burden, measures of benefits and confidence were collected only at first attendance, limiting the ability to assess change over multiple sessions. Finally, the qualitative responses were analysed descriptively in relation to the RQs and should not be interpreted as findings derived from a formal qualitative analytic framework.

## 6 Conclusion

This study demonstrates the feasibility, acceptability, safety and benefits of using a participatory co‐design approach to develop a digital suicide prevention intervention tailored to international students in Australia. Beyond shaping content, the process fostered empowerment, collaboration and shared ownership, strengthened cross‐sector relationships and created a space where students from diverse cultural backgrounds could find common ground and learn from one another. Importantly, this study advances the evidence base by applying theoretically informed measures and a pre–post design to quantify changes in co‐design confidence and empowerment, offering rare empirical data on co‐design outcomes. Despite challenges such as format differences and limited longitudinal assessment, this project provides a promising model for inclusive intervention development. Future research should extend this work by examining longitudinal impacts, scaling to other culturally diverse populations and further leveraging co‐design as a vehicle for collective sense‐making and community building.

## Author Contributions

**Christina Ng:** conceptualization, methodology, investigation, formal analysis, data curation, writing original draft, writing review and editing, project administration. **Tanvi Shaikh:** writing – review and editing, investigation. **Jo Robinson:** conceptualization, methodology, funding acquisition, writing – review and editing. **Jennifer Nicholas:** conceptualization, methodology, writing – review and editing, funding acquisition. **Vivienne Browne:** conceptualization, methodology, funding acquisition, writing – review and editing. **Gina Chinnery:** conceptualization, methodology, funding acquisition, writing – review and editing. **Isabella Choi:** conceptualization, methodology, funding acquisition, writing – review and editing. **Bailey Nation‐Ingle:** conceptualization, methodology, writing – review and editing. **Kristal Allison:** conceptualization, methodology, writing – review and editing. **Ella Perlow:** conceptualization, methodology, writing – review and editing. **Michelle Lamblin:** conceptualization, methodology, funding acquisition, writing – review and editing. **Ellie Brown:** writing – review and editing, supervision. **Gregory Armstrong:** conceptualization, methodology, funding acquisition, writing – review and editing. **Madhavan Mani:** resources, methodology. **Samuel McKay:** conceptualization, methodology, software, data curation, investigation, formal analysis, supervision, resources, project administration, funding acquisition, writing – review and editing.

## Ethics Statement

Ethical approval for this study was obtained from the University of Melbourne Human Research Ethics Committee (approval number: 30125), and the study complied with the Declaration of Helsinki.

## Consent

All participants provided written informed consent.

## Conflicts of Interest

Dr Mani was contracted and paid to develop the Bud app created as part of this study, but had no role in selecting the outcomes, analysing the data or interpreting the results.

## Supporting information

Supporting File 1

Supporting File 2

Supporting File 3

Supporting File 4

Supporting File 5

Supporting File 6

## Data Availability

Research data are not shared due to the ethical constraints of the study.
