# Supplementary material for: A Pre–Post Study of the Feasibility, Acceptability and Benefits of a Co‐Design Approach for the Development of a Digital Suicide Prevention App for International Students
Source: Health Expect. 2026 Apr 13;29(2):e70669. doi: 10.1111/hex.70669 (PMC13074425; doi:10.1111/hex.70669)
Supplement: Supplementary file 2 — Supporting File 2 [file HEX-29-e70669-s006.docx]

# Appendix 1: Workshop Plan

**Table 1.** International student workshop sessions

| **Workshops** | **Stage** | **Content & Activities** | **Outcomes** |
| --- | --- | --- | --- |
| 1 | Understanding Experiences | Journey mapping; Empathy mapping; Digital day in the life | Detailed information on student experiences, potential intervention targets and methods |
| 2 | Understanding Experiences | Existing strengths identification task; Resource and support card sort; What’s shared and what’s unique | Detailed information on student strengths and resources that could be leveraged, potential areas for cross cutting intervention components and cultural modifications |
| 3 & 4 | Exploring ideas | 10 x 10 idea generation exercise; Overview of what we know; Lightning Decision Jam | Identification of preferred problems & solutions |
| 5 | Selecting concepts & Converging | 5 Whys; User persona development; What’s been done before; Best approach | Root causes of problems and best approach for prototype development, foundational considerations for implementation |
| 6 | Converging & Prototyping | What’s been done before; Low fidelity prototyping | Low fidelity prototypes based on decided approach |
| 7 | Prototyping | Prototyping review and refinement; User flow mapping; Toolkit refinement | Feedback on designs, development of different flows through the app |
| 8 | Prototyping & Evaluating | Names & design review; Prototype and content refinement | Feedback on designs, confirmation of direction, and further refinement of content |
| 9 | Evaluating | Design elements review; Prototype and content refinement | Locked in design elements, main content, and user flows |
| 10 | Evaluating | High fidelity prototype review & testing; Implementation planning | Feedback and testing of prototype, implementation & roll-out planning |

**Table 2.** Sector stakeholder workshop sessions

| **Workshops** | **Stage** | **Content & Activities** | **Outcomes** |
| --- | --- | --- | --- |
| 1 | Understanding Experiences | Supports and stakeholder mapping; Mental health challenges of international students mapped against supports; What works and what doesn’t, Opportunities and gaps | Comprehensive map of the stakeholder ecosystem, prioritised list of mental health challenges, detailed inventory of resources and gaps, list of intervention areas |
| 2 | Exploring ideas | Student strengths and resources; Cross-cultural support needs analysis; Cultural sensitivities, Barrier and facilitators | List of student strengths and resources, matrix of shared and unique support needs, guidelines for culturally sensitive support strategies, detailed list of potential barriers and facilitators for implementation |
| 3 | Prototyping and evaluating | Prototype review and feedback; Implementation strategy development | Feedback and testing of prototype, implementation and roll-out planning |
