# Supplementary material for: A Pre–Post Study of the Feasibility, Acceptability and Benefits of a Co‐Design Approach for the Development of a Digital Suicide Prevention App for International Students
Source: Health Expect. 2026 Apr 13;29(2):e70669. doi: 10.1111/hex.70669 (PMC13074425; doi:10.1111/hex.70669)
Supplement: Supplementary file 3 — Supporting File 3 [file HEX-29-e70669-s005.docx]

**Appendix 3: The Digital Intervention**

Bud is a self-guided mobile application co-designed with international students to address proximal drivers of suicide risk in culturally responsive ways. The program focuses on strengthening protective factors and reducing key psychological contributors to suicidality at a community level, rather than providing clinical treatment. Bud adopts a transdiagnostic approach targeting mechanisms associated with psychological distress and suicide risk, including emotion regulation difficulties, perceived burdensomeness, low belonging, maladaptive thinking patterns, and stress-related coping deficits^22,23^.

The app was designed to support international students’ wellbeing by addressing modifiable risk factors such as stress, emotion dysregulation, and social isolation while strengthening protective factors including social support, coping skills, and help-seeking behaviours. Culturally responsive design features include non-clinical framing, diverse student narratives, and peer-oriented content. The app includes:

- a mood-tracking feature that recommended contextually relevant tools drawn from acceptance and commitment therapy (ACT), cognitive behavioural therapy (CBT), and dialectical behaviour therapy (DBT) (e.g., cognitive reframing, breathing exercises, temperature-based distress tolerance strategies, structured problem-solving, and values identification);
- a brief (~20 minute) suicide prevention skills course providing practical guidance on recognising warning signs, initiating supportive conversations, asking directly about suicide, and connecting someone to professional support; and
- narrative stories from international students describing experiences of distress, suicidal thoughts, and help-seeking.

Bud was not designed as a crisis-support tool; users are directed to appropriate support services when needed. Figure 1 provides example screenshots.

Figure 1. Bud app screenshots


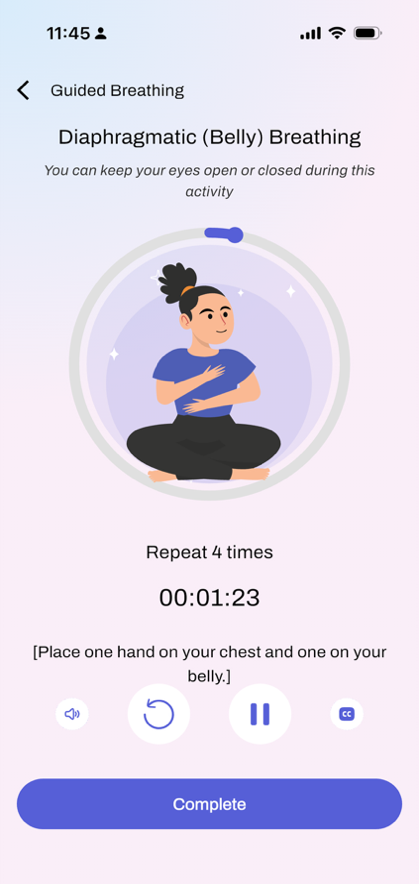

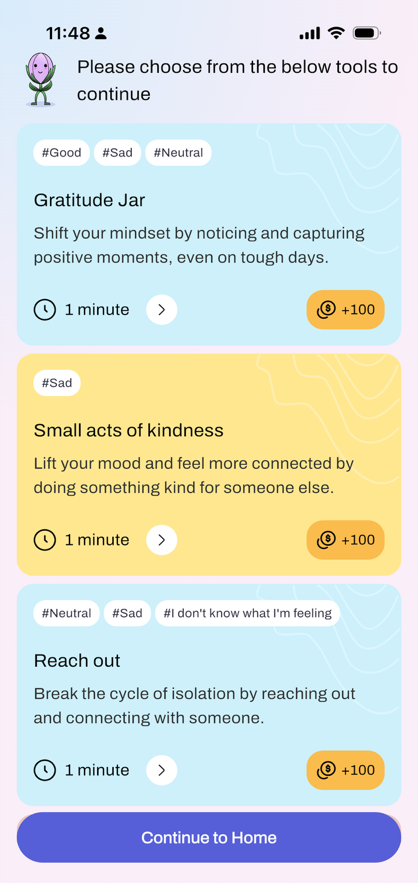

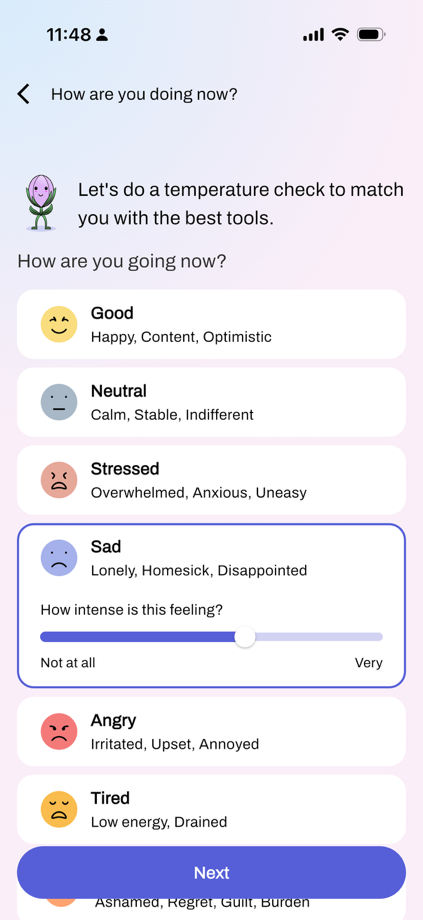

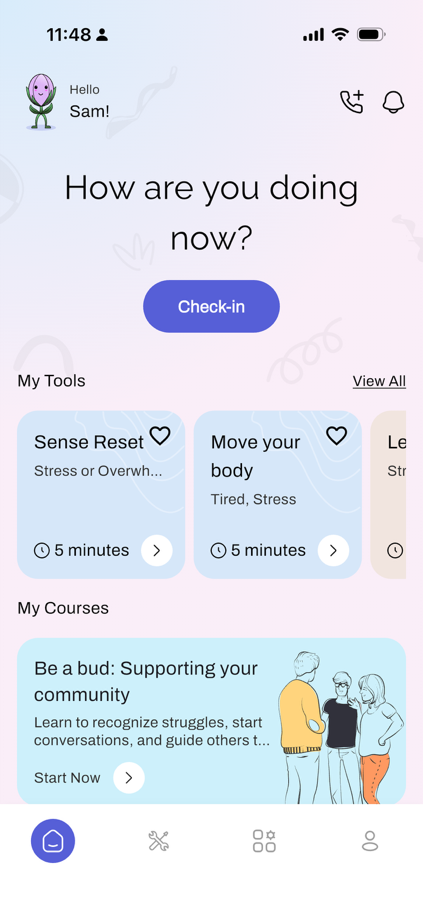

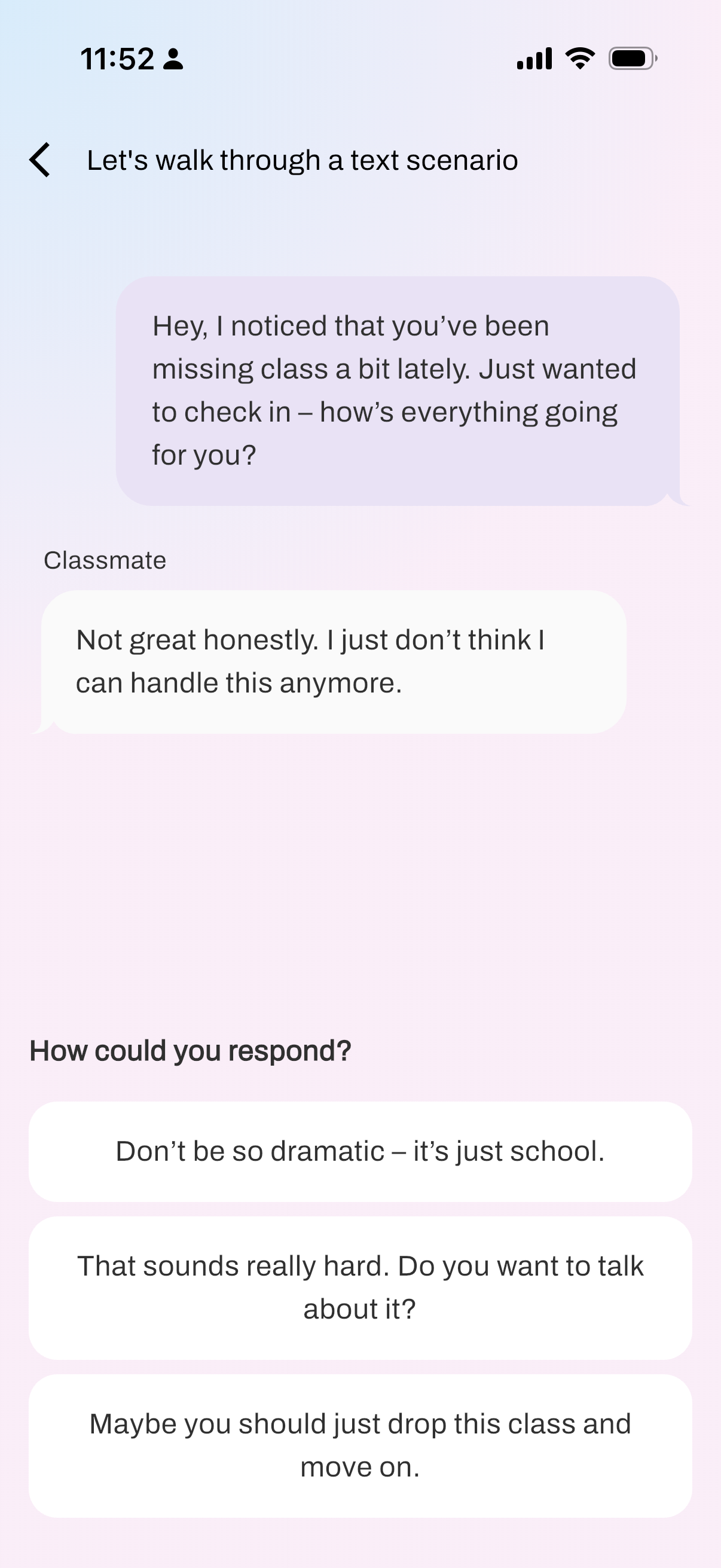

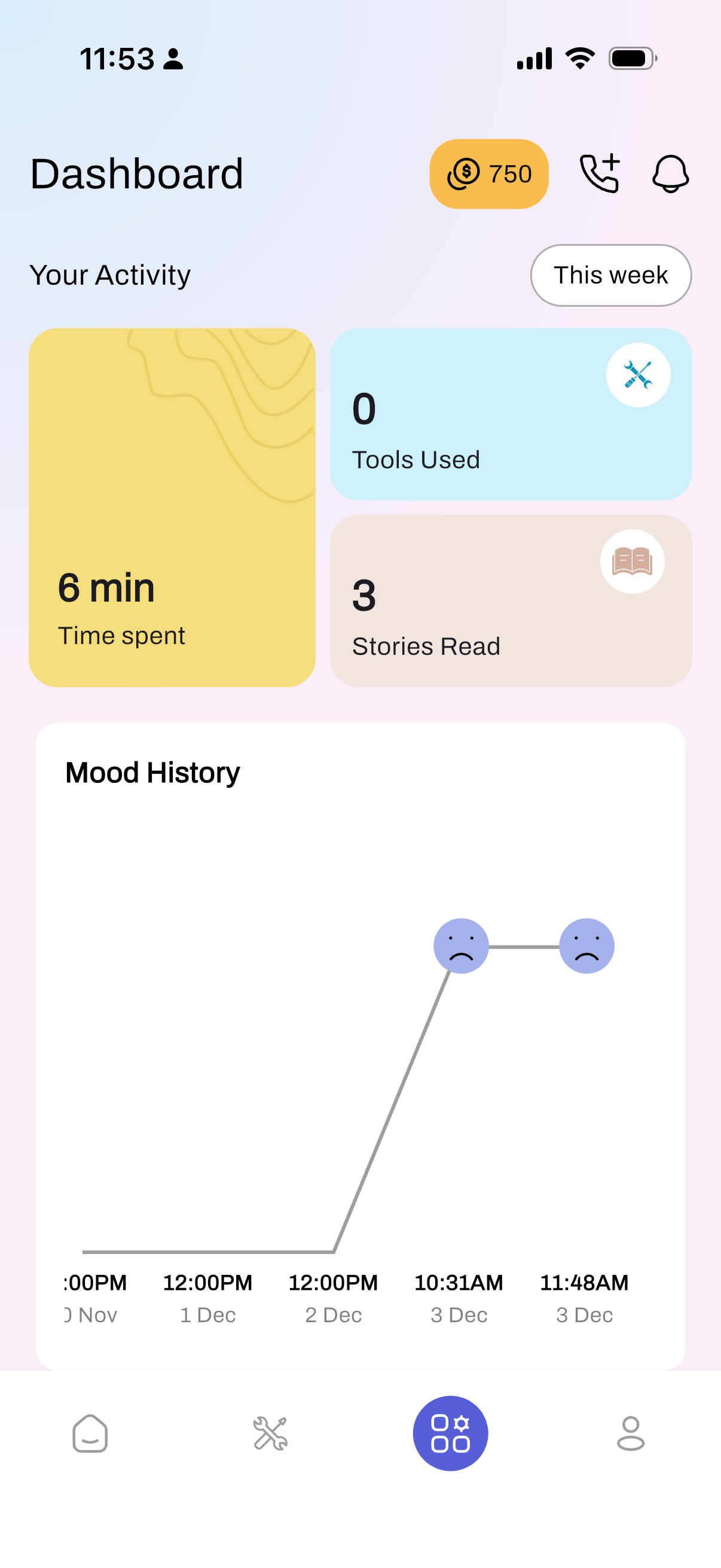


Home page

Mood check-in

Tools

Dashboard

Suicide prevention course

Breathing exercise
