# Supplementary material for: A Pre–Post Study of the Feasibility, Acceptability and Benefits of a Co‐Design Approach for the Development of a Digital Suicide Prevention App for International Students
Source: Health Expect. 2026 Apr 13;29(2):e70669. doi: 10.1111/hex.70669 (PMC13074425; doi:10.1111/hex.70669)
Supplement: Supplementary file 4 — Supporting File 4 [file HEX-29-e70669-s002.docx]

# Appendix 2: Supporting Documents for Student Workshops

# Helpful definitions

**Co-design workshop**: a workshop where you help create and improve a program

**Cultural competence**: Ability to understand and communicate with people across different cultures

**Cultural/Ethnic groups**: e.g., South East Asian, Australian, Northern European, South Asian

**Distress**: Suffering of body or mind e.g., pain, upset, worried, troubled, disturbed, anxious

**Gender Identity**: What you personally identify as e.g., male, female or another gender

**Help-seeking**: Communicating problems or issues or to get support, advice or help on those problems or issues

**Implementation**: To carry out a plan or idea or to put a plan into action

**Intervention**: Providing support or doing something to improve a situation or prevent it from getting worse (e.g., web page that provides information and wellbeing or mental health support to international students)

**Lived experiences**: Your personal experience

**Mental health literacy**: Knowledge and beliefs about mental disorders, such as how recognise, manage or prevent problems

**Mental health**: State of mental well-being that allows people to cope and handle stress, relate to others, learn and work well

**Nationality**: Citizenship or country you are from

**Native language**: The first language you learnt from birth

**Power differences**: Differences in authority or control between people e.g., Older – younger, Professor – Student, Boss/Manager – Employee

**Prevention**: The act of stopping something from happening or stopping someone from doing something e.g., preventing crime

**Protective factors**: Something that reduces the possibility that a bad thing will happen e.g., feeling connected with others or having support from other people can improve mental health

**Risk factors**: Something that increases the possibility that a bad thing will happen e.g., loneliness or isolation can increase your risk to having suicidal thoughts

**Self-harm**: Hurting yourself on purpose

**Self-soothing tools**: Things that help you feel calm

**Sex**: Label that you were assigned to at birth e.g., male or female

**Sexuality**: Who you are attracted to

**Spiritual beliefs:** Your religion or other belief systems e.g., Islam, Buddhism etc

**Stigma:** A set of negative beliefs that people or a group of people can have about someone or something that can be unfair or false e.g., stigma of mental illness

**Suicidal ideation**: Thoughts about dying by suicide

**Suicide**: When a person takes his or her own life, or does something to make himself or herself die

**Transgender/gender diverse**: Someone whose gender identity is different from the sex they were assigned at birth

**Wellbeing**: State of feeling comfortable, happy and healthy

**Safe language guide**

| Don’t say | Do say | Why |
| --- | --- | --- |
| 'committed' or 'commit suicide' | 'died by suicide' or 'ended his/her/their own life' | To avoid association between suicide and 'crime' or 'sin' that may alienate some people. |
| 'unsuccessful suicide' | 'non-fatal' or 'made an attempt on his/her/their life' | To avoid presenting suicide as a desired outcome or glamourising a suicide attempt. |
| 'successful suicide' | 'took their own life', 'died by suicide' or 'ended their own life' | To avoid presenting suicide as a desired outcome. |
| 'suicide epidemic' | 'concerning rates of suicide' | To avoid sensationalism and inaccuracy. |
| 'mental patient', 'nutter', 'lunatic', 'psycho', 'schizo', 'deranged', 'mad' | A person is 'living with' or 'has a diagnosis of' mental illness. | Certain language sensationalises mental illness and reinforces stigma. |
| 'victim', 'suffering from' or 'affected with' a mental illness | A person is 'being treated for', or 'someone with a mental illness'. | Terminology that suggests a lack of quality of life for people with mental illness. |
| A person is 'a schizophrenic', 'an anorexic' | A person 'has a diagnosis of' or 'is being treated for...' | Labelling a person by their mental illness. |
| 'crazed', 'deranged', 'mad', 'psychotic' | The person's behaviour was unusual or erratic. | Descriptions of behaviour that imply existence of mental illness or are inaccurate. |
| 'happy pills', 'shrinks', 'mental institution' | Antidepressants, psychiatrists or psychologists, mental health hospital. | Colloquialisms about treatment can undermine people's willingness to seek help. |
| 'psychotic dog', using 'schizophrenic' to denote duality such as 'schizophrenic economy'. | Reword any sentence that uses psychiatric or medical terminology incorrectly or out of context. | Terminology used out of context adds to misunderstanding and trivialises mental illness. |

Extracts from Roses in the Ocean Language and Imagery Guide: <https://rosesintheocean.com.au/wp-content/uploads/2022/09/RITO-Language-Imagery-Guide-for-LESEPI-v1.2-spread-for-digital-viewing.pdf>
