# Supplementary material for: A Pre–Post Study of the Feasibility, Acceptability and Benefits of a Co‐Design Approach for the Development of a Digital Suicide Prevention App for International Students
Source: Health Expect. 2026 Apr 13;29(2):e70669. doi: 10.1111/hex.70669 (PMC13074425; doi:10.1111/hex.70669)
Supplement: Supplementary file 5 — Supporting File 5 [file HEX-29-e70669-s004.docx]

# Appendix 3: Study Measures

# MEASURES FOR STUDENTS Time 1

Thank you for choosing to participate in a co-design workshop to help develop the new digital international student suicide prevention program. This survey will ask you questions about your background and experiences with suicide. It will take you approximately 15 minutes to complete.

These surveys contain references to suicide and/or self-harm. If you feel like you need support, please reach out to the facilitator in the room. If you feel concerned or distressed after the workshop, you can contact the services below:

| **eheadspace** | **Kids Helpline** | **Lifeline** |
| --- | --- | --- |
| 12-25 years | 5-25 years | All ages |
| 9:00am-1:00am AEST | 24/7 | 24/7 |
| headspace.org.au/online-and-phone-support | kidshelpline.com.au | lifeline.org.au |
| 1800 650 890 | 1800 55 1800 | 13 11 14 |

You can also save these numbers in case you need them.

## Demographics

1. **Is this your first time completing a co-design workshop for this project?**

- Yes
- No *–* Please enter the code provided by the researcher: ___________ *Skips to Q26*

1. **What is your date of birth? (Quantitative)**

[Please enter text here]

1. **What is your gender identity?  (Qualitative)**

[Please enter text here]

1. **Do you identify as transgender or gender diverse? (Qualitative)**

- Yes
- No
- Questioning/Unsure
- Prefer not to disclose

1. **At birth, was your sex recorded as: (Qualitative)**

- Male
- Female
- Another term (please specify): ___________
- Prefer not to disclose

1. **How would you define or describe your sexuality? (Qualitative)**

- Heterosexual/straight
- Lesbian
- Gay
- Bisexual
- Pansexual
- Queer
- Questioning/Not Sure
- Not listed (please specify):
- Prefer not to disclose

1. **What is your nationality (Qualitative)**

[Please enter text here]

1. **If any, what cultural or ethnic groups do you most identify with (e.g. Maori, Greek, Malaysian, Filipino, Australian)? (Open-ended) (Qualitative)**

[Please enter text here]

1. **If any, what religious, spiritual or other set of beliefs do you most identify with (e.g. Islam, Buddhism, Hinduism, Sikhism, Christianity, Atheism)? (Open-ended) (Qualitative)**

[Please enter text here]

1. **How long have you lived in Australia? (Qualitative)**

[Please enter text here – specified in months and years]

1. **Which country have you lived in for the majority of your life? (Qualitative)**

[Please enter text here]

1. **How long did you live in this country? (Qualitative)**

[Please enter text here – specified in months and years]

1. **What language do you speak most of the time? (Qualitative)**

- English
- Other [Please enter text here]

1. **What is your native language? (Qualitative)**

[Please enter text here]

1. **Are you currently studying? (Quantitative)**

- Yes:

**What is the name of your educational institution?**

[Please enter text here]

**What is your major?**

[Please enter text here]

- No

1. **Please indicate your highest level of completed education: (Qualitative)**

- Year 7 - 8 (junior high school)
- Year 9 – 10 (middle high school)
- Year 11 - 12 (senior high school)
- Some university
- Some TAFE
- Completed bachelor’s degree
- Completed TAFE course/certificate
- Completed post-graduate degree
- Other (please specify):

1. **What is your current employment status? (Qualitative)**

- Employed – Casual
- Employed – Part time
- Employed – Full time
- Employed – Studying and Working
- Full time student
- Looking for work
- Unable to work
- Other (please describe):
- Prefer not to disclose

## Suicide ideation/Self-harm (Qualitative)

This section will ask you some questions about your own experiences with self-harm and suicide.

Before you continue any further, let's take a moment to check-in. This questionnaire contains items related to self-harm and suicide. For some people, these questions can feel difficult and may bring up unpleasant feelings or memories. It is important that you take care of yourself and keep yourself safe while completing this questionnaire.

You can take care of yourself by:

1. Asking yourself if you are ready to focus on this topic (it is okay if you are not)
2. Having someone sit with you (in-person, online, or via phone) while you complete it

3) Having some self-soothing tools nearby and strategies prepared (e.g., sensory toys; such as plush toys or squishy toys, your favourite music, grounding activities etc.) Here are some examples:

5-5-5 Breathing exercise

- Breathe in deeply for 5 seconds, hold your breath for 5 seconds, then breathe out for 5 seconds

5-4-3-2-1 Senses Activity

- Focus on;
  - 5 things you can **see**
  - 4 things you can physically **feel**
  - 3 things you can **hear**
  - 2 things you can **smell**
  - 1 thing you can **taste**

4) Planning something nice to do after completing the questionnaire or sections of the questionnaire

5) Taking regular breaks

6) Letting someone you trust know before you start completing it and check-in with them after you've completed it
7) Reaching out to people and places who can support you.

If it feels too much and you feel unable to stay in the questionnaire and complete it, please exit the questionnaire, and reach out to people and places who will be able to provide you with support. You can save this survey and return later if you would like. There will be no consequences for withdrawal. Your safety is our highest priority.

1. **In your lifetime, have you ever had thoughts of hurting yourself?**

- Yes
- No

1. **In your lifetime, have you ever engaged in self-harm?**

- Yes
- No

1. **In your lifetime, have you ever had suicidal thoughts (for example, thoughts that you would be better off dead or thoughts of killing yourself)?**

- Yes
- No

1. **In your lifetime, have you ever made a suicide attempt?**

- Yes
- No

1. **Have you ever experienced a suicide death of someone that you know (e.g., a friend, a sibling)?**

- Yes
- No

1. **What was your relationship to this person?**

- Brother
- Sister
- Friend
- Workmate
- Someone I knew through my online community
- Friend of a friend
- Other family member
- Other
- Rather not say

1. **Please specify: How long ago did this suicide occur?**

- In the past month
- In the past 6 months
- In the past year
- In the past two years
- In the past three years
- More than four years ago

## Mid-survey check in

Thank you for answering these questions so far, we know it can be difficult to do so and really appreciate your contributions so far.

If you would like to take a moment to pause from this survey, please click the ‘Save & Return Later’ button down the bottom.

If you would like to talk to someone, please reach out to any of the following services:

| **eheadspace** | **Kids Helpline** | **Lifeline** |
| --- | --- | --- |
| 12-25 years | 5-25 years | All ages |
| 9:00am-1:00am AEST | 24/7 | 24/7 |
| headspace.org.au/online-and-phone-support | kidshelpline.com.au | lifeline.org.au |
| 1800 650 890 | 1800 55 1800 | 13 11 14 |

It might be a **good idea to take a screenshot of these numbers**, so you always have them available to you.

The next few questions will ask you about your feelings towards co-design and your reasons for participating in this workshop, as well as your current confidence levels in communicating safely online about self-harm and suicide.

## Acceptability (Meeting Expectations)

1. **What made you want to be involved with the co-design project? (Qualitative)**

[Please enter text here]

1. **What do you hope to get out of this co-design workshop? Please select as many as apply (Qualitative)**

- Opportunity for my voice to be heard.
- Be involved in improving self-harm and suicide prevention research.
- Opportunity to advocate for support for international students in my community.
- Better understand self-harm and suicide prevention research.
- Better understand online tools could prevent suicide among international students.
- Work collaboratively with others.
- Connect socially with others.
- Gain understanding about how co-design can help improve self-harm and suicide prevention outcomes.
- Opportunities to connect with professional researchers and designers.
- I have no expectations for the co-design workshop.
- Other: [Please enter text here]

## Skills

| **How confident are you in your ability to do the following during a co-design workshop:** | Not at all confident | Not very confident | Neutral | Confident | Very confident |
| --- | --- | --- | --- | --- | --- |
| 1. **Understand the workshop goals** |  |  |  |  |  |
| 1. **Meaningfully contribute** |  |  |  |  |  |
| 1. **Work collaboratively** |  |  |  |  |  |
| 1. **Receive and incorporate feedback from others** |  |  |  |  |  |
| 1. **Adapt to changes or new directions** |  |  |  |  |  |
| 1. **Participate in co-design activities (e.g., brainstorming, prototyping)** |  |  |  |  |  |
| 1. **Impact decisions or project outcomes** |  |  |  |  |  |
| 1. **Handle power differences** |  |  |  |  |  |
| 1. **Care for your well-being** |  |  |  |  |  |
| 1. **Seek support if you need it** |  |  |  |  |  |
| 1. **Share your lived experiences in a way that feels safe and respectful** |  |  |  |  |  |

1. **Is there any feedback you would like to provide about participating in this research or completing the survey?**

[Please enter text here]

# Time 2

Thank you for taking this survey. It will take approximately 20 minutes to complete.

Before you begin, please note that these surveys contain references to suicide and/or self-harm. If you feel like you need support, please reach out to the facilitator in the room. If you feel concerned or distressed after leaving the workshop, the following services are available:

| **eheadspace** | **Kids Helpline** | **Lifeline** |
| --- | --- | --- |
| 12-25 years | 5-25 years | All ages |
| 9:00am-1:00am AEST | 24/7 | 24/7 |
| headspace.org.au/online-and-phone-support | kidshelpline.com.au | lifeline.org.au |
| 1800 650 890 | 1800 55 1800 | 13 11 14 |

It might also be a good idea to save these numbers so that you have them on hand, should you need them.

Please press "Start" to begin the survey.

## Funder Required Questions

1. **Are you an international student currently studying with Victorian education or training provider?**

- I am considering study as an international student with a Victorian education or training provider
- I am a current international student enrolled with a Victorian education or training provider
- I have previously studied as an international student with a Victorian education or training provider
- None of the above (if selected, do not ask question 2)

1. **Overall, how satisfied or dissatisfied were you with the international student suicide prevention co-design workshop, delivered through the Study Melbourne Inclusion Program by Orygen?**

- Very dissatisfied
- Dissatisfied
- Neither satisfied nor dissatisfied
- Satisfied
- Very satisfied

## Feasibility

Please rate how strongly you agree or disagree with the following statements. **(Quantitative)**

|  | Strongly Disagree | Disagree | Neither | Agree | Strongly Agree |
| --- | --- | --- | --- | --- | --- |
| 1. **It was easy to participate in this workshop** |  |  |  |  |  |
| 1. **I felt supported to participate in this workshop** |  |  |  |  |  |
| 1. **It was convenient to participate in this workshop** |  |  |  |  |  |
| 1. **I was fairly paid for my time to participate in this workshop** |  |  |  |  |  |

1. **Please describe why you agreed or disagreed with the above statements here (Optional): (Qualitative)**

[Please enter text here]

## Acceptability

**Please indicate below to what extent you agree or disagree with the following statements (Quantitative)**

|  | Strongly disagree | Disagree | Neutral | Agree | Strongly Agree |
| --- | --- | --- | --- | --- | --- |
| 1. **I felt included in the co-design process** |  |  |  |  |  |
| 1. **My needs were met in the co-design process** |  |  |  |  |  |
| 1. **Participating in the co-design workshop was useful** |  |  |  |  |  |
| 1. **Participating in co-design workshop was worth the time and effort** |  |  |  |  |  |
| 1. **I felt I connected socially and meaningfully with other participants in this workshop** |  |  |  |  |  |
| 1. **A diverse range of voices and experiences were included in the workshop** |  |  |  |  |  |
| 1. **The workshop was representative of my opinions** |  |  |  |  |  |
| 1. **Power was distributed fairly between participants and researchers** |  |  |  |  |  |
| 1. **All participants had equal opportunities to share their ideas and perspectives** |  |  |  |  |  |
| 1. **The facilitators created a supportive and emotionally safe environment** |  |  |  |  |  |
| 1. **The cultural backgrounds and values of all participants were respected and included** |  |  |  |  |  |
| 1. **I felt that my expertise was valued and utilised in the workshop** |  |  |  |  |  |
| 1. **I had access to all the resources and information I needed to participate fully** |  |  |  |  |  |
| 1. **In the previous survey you indicated that your expectations for the workshop were *[piped responses from Q25 in T1 survey]*. Did the workshop meet these expectations?** |  |  |  |  |  |

1. **Please describe why you agreed or disagreed with the above statements here (Optional). (Qualitative)**

[Please enter text here]

1. **Overall, to what degree did you enjoy the co-design workshop? (Qualitative)**

- Did not enjoy at all
- Mostly did not enjoy
- Neutral
- Mostly enjoyed
- Enjoyed it all

**Please rate the degree to which you were satisfied/enjoyed the following aspects of the co-design workshop. (Quantitative)**

|  | Not satisfied/Did not enjoy |  |  |  | Very satisfied/Enjoyed |
| --- | --- | --- | --- | --- | --- |
|  | 1 | 2 | 3 | 4 | 5 |
| 1. **Facilitators** |  |  |  |  |  |
| 1. **Design activities** |  |  |  |  |  |
| 1. **Connecting socially with other participants** |  |  |  |  |  |
| 1. **Working collaboratively with other participants** |  |  |  |  |  |
| 1. **Connecting with professionals in the field** |  |  |  |  |  |
| 1. **Working on the new intervention** |  |  |  |  |  |
| 1. **Learning about suicide prevention** |  |  |  |  |  |
| 1. **Resources available** |  |  |  |  |  |

1. **If we were to run this co-design workshop again, what would you like to see improved or done differently? Please be as detailed as you can (Qualitative)**[Please enter text here]

## Acceptability (Safety) (Quantitative)

Please rate each of the following statements (1 – Strongly Disagree ; 5 – Strongly Agree )

1. **The co-design workshop made me feel upset**
2. **The co-design workshop made me feel suicidal**
3. **The co-design workshop made me want to self-harm**
4. **I felt emotionally/psychologically safe* taking part in the co-design workshop**
5. **I felt culturally safe* taking part in the co-design workshop**

If you are unsure what some of these terms mean, please click on the following terms for some definitions:

*Culturally Safe Definition*

“An environment that is safe for people: where there is no assault, challenge or denial of their identity, of who they are and what they need. It is about shared respect, shared meaning, shared knowledge and experience of learning, living and working together with dignity and truly listening.” - Australian Human Rights Commission, Social Justice Report 2011

*Emotionally/Psychologically Safe Definition*

“A feeling or climate whereby the learner [you] can feel valued and comfortable yet still speak up and take risks without fear of retribution, embarrassment, judgment or consequences either to themselves or others, thereby promoting learning and innovation.” - Turner et al. 2018

*NOTE: If a rating of 4 or 5 for questions 26, 27 or 28; and/or rating of 1 or 2 for questions 29 or 30 were selected, RESEARCHERS NOTIFIED AND RISK MANAGEMENT PLAN ENACTED*

## Benefits for international students participating in the workshops

Please rate the following statements (Quantitative)

| *As a results of the co-design workshop:* | Strongly disagree | Disagree | Neutral | Agree | Strongly Agree |
| --- | --- | --- | --- | --- | --- |
| 1. **I know more about research** |  |  |  |  |  |
| 1. **I know more about suicide prevention** |  |  |  |  |  |
| 1. **I know more about mental health** |  |  |  |  |  |
| 1. **I am more aware of available mental health support services** |  |  |  |  |  |
| 1. **I feel that I can contribute to suicide prevention initiatives** |  |  |  |  |  |
| 1. **I feel empowered to improve my own mental health** |  |  |  |  |  |
| 1. **I feel empowered to support others to improve their mental health** |  |  |  |  |  |
| 1. **I feel a greater sense of belonging** |  |  |  |  |  |
| 1. **I feel more socially connected** |  |  |  |  |  |
| 1. **I feel like my experience as an international student is valued** |  |  |  |  |  |
| 1. **I am more likely to seek professional help if experiencing mental health problems** |  |  |  |  |  |
| 1. **I am more likely to talk to my friends about their mental health** |  |  |  |  |  |
| 1. **I am more likely to recommend my friends seek professional help for their mental health problems** |  |  |  |  |  |

## **Skills (Quantitative)**

| **How confident are you in your ability to do the following during a co-design workshop:** | Not at all confident | Not very confident | Neutral | Confident | Very confident |
| --- | --- | --- | --- | --- | --- |
| 1. **Understand the workshop goals** |  |  |  |  |  |
| 1. **Meaningfully contribute** |  |  |  |  |  |
| 1. **Work collaboratively** |  |  |  |  |  |
| 1. **Receive and incorporate feedback from others** |  |  |  |  |  |
| 1. **Adapt to changes or new directions** |  |  |  |  |  |
| 1. **Participate in co-design activities (e.g., brainstorming, prototyping)** |  |  |  |  |  |
| 1. **Impact decisions or project outcomes** |  |  |  |  |  |
| 1. **Handle power differences** |  |  |  |  |  |
| 1. **Care for your well-being** |  |  |  |  |  |
| 1. **Seek support if you need it** |  |  |  |  |  |
| 1. **Share your lived experiences in a way that feels safe and respectful** |  |  |  |  |  |

1. **What was the most valuable part of the workshop for you? (Qualitative)**

[Please enter text here]

1. **Which specific co-design skills have you improved through this workshop? (Qualitative)**

[Please enter text here]

1. **Would you like to do any of the following?**

- Complete an interview about your experience of the co-design workshop (reimbursed $30 for your time)
- Attend future co-design workshops to develop the new intervention further
- Test or provide feedback on the prototype intervention
- Receive updates on the current project progress
- Be kept up to date about future projects or opportunities
- Volunteer to assist with future workshops or events

**If you selected any of the above options, please enter your email**
[Please enter text here]

## Other feedback

**Please leave any feedback you might have for the research team about your experience throughout this co-design workshop or the survey (Qualitative)**[Please enter text here]

## End of Survey

Thank you for completing your survey and for participating in this important research! This now brings you to the end of this part of the research project.

# MEASURES FOR STAKEHOLDERS

# Time 1

Thank you for choosing to participate in a co-design workshop to support the development of a new digital international student suicide prevention program. This survey will ask you questions about your background and experiences with suicide. It will take you approximately 15 minutes to complete.

these surveys contain references to suicide and/or self-harm. If you feel like you need support, please reach out to the facilitator in the room. If you feel concerned or distressed after leaving the workshop, the following services are available:

| **eheadspace** | **Kids Helpline** | **Lifeline** |
| --- | --- | --- |
| 12-25 years | 5-25 years | All ages |
| 9:00am-1:00am AEST | 24/7 | 24/7 |
| headspace.org.au/online-and-phone-support | kidshelpline.com.au | lifeline.org.au |
| 1800 650 890 | 1800 55 1800 | 13 11 14 |

It might also be a good idea to save these numbers so that you have them on hand, should you need them.

## Demographics

1. **Is this your first time completing a co-design workshop for this project?**

- Yes
- No *–* Please enter the code provided by the researcher: ___________ *Skips to Q9*

1. **What is your job title? (Qualitative)**

[Please enter text here]

1. **What organisation do you represent? (Qualitative)**

[Please enter text here]

1. **In your role, how do you interact with international students? (Select all that apply and provide details where applicable) (Quantitative)**

- Administration
- Housing
- Teaching
- Mental health services
- Academic Advising
- Career Services
- Student Affairs
- Counselling Services
- Language Support Services
- Financial Aid and Scholarships
- Cultural and Social Activities Coordination
- Health Services
- Legal and Immigration Support
- Orientation and Transition Programs
- Student Clubs and Organizations
- Tutoring and Academic Support Services
- Community Engagement and Outreach
- IT and Technology Support
- Other (please specify): __________

1. **How many years have you been working in the international education sector? (Qualitative)**

[Please enter text here]

1. **Do you identify as belonging to any underrepresented or minority groups? (e.g., ethnic, racial, LGBTQIA+, persons with disabilities) (Qualitative)**

[Please enter text here]

1. **Are there any specific accommodations you need to fully participate in this workshop? (Qualitative)**[Please enter text here]

## Acceptability (Meeting Expectations)

1. **What made you want to be involved with the co-design project? (Qualitative)**

[Please enter text here]

1. **What do you hope to get out of this co-design workshop? Please select as many as apply (Qualitative)**

- Opportunity for my voice to be heard.
- Be involved in improving self-harm and suicide prevention research.
- Opportunity to advocate for support for international students in my community.
- Better understand self-harm and suicide prevention research.
- Better understand online tools could prevent suicide among international students.
- Work collaboratively with others.
- Connect socially with others.
- Gain understanding about how co-design can help improve self-harm and suicide prevention outcomes.
- Opportunities to connect with professional researchers and designers.
- I have no expectations for the co-design workshop.
- Other: [Please enter text here]

## Skills

| **How confident are you in your ability to do the following during a co-design workshop:** | Not at all confident | Not very confident | Neutral | Confident | Very confident |
| --- | --- | --- | --- | --- | --- |
| 1. **Understand the workshop goals** |  |  |  |  |  |
| 1. **Meaningfully contribute** |  |  |  |  |  |
| 1. **Work collaboratively** |  |  |  |  |  |
| 1. **Receive and incorporate feedback from others** |  |  |  |  |  |
| 1. **Adapt to changes or new directions** |  |  |  |  |  |
| 1. **Participate in co-design activities (e.g., brainstorming, prototyping)** |  |  |  |  |  |
| 1. **Impact decisions or project outcomes** |  |  |  |  |  |
| 1. **Handle power differences** |  |  |  |  |  |
| 1. **Care for your well-being** |  |  |  |  |  |
| 1. **Seek support if you need it** |  |  |  |  |  |
| 1. **Share your lived experiences in a way that feels safe and respectful** |  |  |  |  |  |

1. **Is there any feedback you would like to provide about participating in this research or completing the survey?**

[Please enter text here]

# STAKEHOLDER MEASURES

# Time 2

Thank you for taking this survey. It will take approximately 20 minutes to complete.

Before you begin, please note that these surveys contain references to suicide and/or self-harm. If you feel like you need support, please reach out to the facilitator in the room. If you feel concerned or distressed after leaving the workshop, the following services are available:

| **eheadspace** | **Kids Helpline** | **Lifeline** |
| --- | --- | --- |
| 12-25 years | 5-25 years | All ages |
| 9:00am-1:00am AEST | 24/7 | 24/7 |
| headspace.org.au/online-and-phone-support | kidshelpline.com.au | lifeline.org.au |
| 1800 650 890 | 1800 55 1800 | 13 11 14 |

It might also be a good idea to save these numbers so that you have them on hand, should you need them.

Please press "Start" to begin the survey.

## Feasibility

Please rate how strongly you agree or disagree with the following statements. **(Quantitative)**

|  | Strongly Disagree | Disagree | Neither | Agree | Strongly Agree |
| --- | --- | --- | --- | --- | --- |
| 1. **It was easy to participate in this workshop** |  |  |  |  |  |
| 1. **I felt supported to participate in this workshop** |  |  |  |  |  |
| 1. **It was convenient to participate in this workshop** |  |  |  |  |  |
| 1. **I was fairly paid for my time to participate in this workshop** |  |  |  |  |  |

1. **Please describe why you agreed or disagreed with the above statements here (Optional): (Qualitative)**

[Please enter text here]

## Acceptability

**Please indicate below to what extent you agree or disagree with the following statements (Quantitative)**

|  | Strongly disagree | Disagree | Neutral | Agree | Strongly Agree |
| --- | --- | --- | --- | --- | --- |
| 1. **I felt included in the co-design process** |  |  |  |  |  |
| 1. **My needs were met in the co-design process** |  |  |  |  |  |
| 1. **Participating in the co-design workshop was useful** |  |  |  |  |  |
| 1. **Participating in co-design workshop was worth the time and effort** |  |  |  |  |  |
| 1. **I felt I connected socially and meaningfully with other participants in this workshop** |  |  |  |  |  |
| 1. **A diverse range of voices and experiences were included in the workshop** |  |  |  |  |  |
| 1. **The workshop was representative of my opinions** |  |  |  |  |  |
| 1. **Power was distributed fairly between participants and researchers** |  |  |  |  |  |
| 1. **All participants had equal opportunities to share their ideas and perspectives** |  |  |  |  |  |
| 1. **The facilitators created a supportive and emotionally safe environment** |  |  |  |  |  |
| 1. **The cultural backgrounds and values of all participants were respected and included** |  |  |  |  |  |
| 1. **I felt that my expertise was valued and utilized in the workshop** |  |  |  |  |  |
| 1. **I had access to all the resources and information I needed to participate fully** |  |  |  |  |  |
| 1. **In the previous survey you indicated that your expectations for the workshop were *[piped responses from Q25 in T1 survey]*. Did the workshop meet these expectations for you?** |  |  |  |  |  |

1. **Please describe why you agreed or disagreed with the above statements here (Optional). (Qualitative)**

[Please enter text here]

1. **Overall, to what degree did you enjoy the co-design workshop? (Qualitative)**

- Did not enjoy at all
- Mostly did not enjoy
- Neutral
- Mostly enjoyed
- Enjoyed it all

**Please rate the degree to which you were satisfied/enjoyed the following aspects of the co-design workshop. (Quantitative)**

|  | Not satisfied/Did not enjoy |  |  |  | Very satisfied/Enjoyed |
| --- | --- | --- | --- | --- | --- |
|  | 1 | 2 | 3 | 4 | 5 |
| 1. **Facilitators** |  |  |  |  |  |
| 1. **Design activities** |  |  |  |  |  |
| 1. **Connecting socially with other participants** |  |  |  |  |  |
| 1. **Working collaboratively with other participants** |  |  |  |  |  |
| 1. **Connecting with professionals in the field** |  |  |  |  |  |
| 1. **Working on the new intervention** |  |  |  |  |  |
| 1. **Learning about suicide prevention** |  |  |  |  |  |
| 1. **Resources available** |  |  |  |  |  |

1. **If we were to run this co-design workshop again, what would you like to see improved or done differently? Please be as detailed as you can (Qualitative)**[Please enter text here]

## Acceptability (Safety) (Quantitative)

Please rate each of the following statements (1 – Strongly Disagree ; 5 – Strongly Agree )

1. **The co-design workshop made me feel upset**
2. **The co-design workshop made me feel suicidal**
3. **The co-design workshop made me want to self-harm**
4. **I felt emotionally/psychologically safe* taking part in the co-design workshop**
5. **I felt culturally safe* taking part in the co-design workshop**

If you are unsure what some of these terms mean, please click on the following terms for some definitions:

*Culturally Safe Definition*

“An environment that is safe for people: where there is no assault, challenge or denial of their identity, of who they are and what they need. It is about shared respect, shared meaning, shared knowledge and experience of learning, living and working together with dignity and truly listening.” - Australian Human Rights Commission, Social Justice Report 2011

*Emotionally/Psychologically Safe Definition*

“A feeling or climate whereby the learner [you] can feel valued and comfortable yet still speak up and take risks without fear of retribution, embarrassment, judgment or consequences either to themselves or others, thereby promoting learning and innovation.” - Turner et al. 2018

*NOTE: If a rating of 4 or 5 for questions 26, 27 or 28; and/or rating of 1 or 2 for questions 29 or 30 were selected, RESEARCHERS NOTIFIED AND RISK MANAGEMENT PLAN ENACTED*

## Skills (Quantitative)

| **How confident are you in your ability to do the following during a co-design workshop:** | Not at all confident | Not very confident | Neutral | Confident | Very confident |
| --- | --- | --- | --- | --- | --- |
| 1. **Understand the workshop goals** |  |  |  |  |  |
| 1. **Meaningfully contribute** |  |  |  |  |  |
| 1. **Work collaboratively** |  |  |  |  |  |
| 1. **Receive and incorporate feedback from others** |  |  |  |  |  |
| 1. **Adapt to changes or new directions** |  |  |  |  |  |
| 1. **Participate in co-design activities (e.g., brainstorming, prototyping)** |  |  |  |  |  |
| 1. **Impact decisions or project outcomes** |  |  |  |  |  |
| 1. **Handle power differences** |  |  |  |  |  |
| 1. **Care for your well-being** |  |  |  |  |  |
| 1. **Seek support if you need it** |  |  |  |  |  |
| 1. **Share your lived experiences in a way that feels safe and respectful** |  |  |  |  |  |

1. **What was the most valuable part of the workshop for you? (Qualitative)**

[Please enter text here]

1. **Which specific co-design skills have you improved through this workshop? (Qualitative)**

[Please enter text here]

1. **Would you like to do any of the following?**

- Complete an interview about your experience of the co-design workshop
- Attend future co-design workshops to develop the new intervention further
- Test or provide feedback on the prototype intervention
- Receive updates on the project’s progress
- Be kept up to date about future projects or opportunities
- Volunteer to assist with future workshops or events

**If you selected any of the above options, please enter your email**
[Please enter text here]

## Other feedback

**Please leave any feedback you might have for the research team about your experience throughout this co-design workshop or the survey (Qualitative)**[Please enter text here]

## End of Survey

Thank you for completing your survey and for participating in this important research! This now brings you to the end of this component of the research project.
