# Supplementary material for: A Pre–Post Study of the Feasibility, Acceptability and Benefits of a Co‐Design Approach for the Development of a Digital Suicide Prevention App for International Students
Source: Health Expect. 2026 Apr 13;29(2):e70669. doi: 10.1111/hex.70669 (PMC13074425; doi:10.1111/hex.70669)
Supplement: Supplementary file 6 — Supporting File 6 [file HEX-29-e70669-s001.docx]

# Supplementary Tables

**Supplementary Table 1.** Detailed student participant demographic data

| **Variable** | **N** | **Proportion** |
| --- | --- | --- |
| ***Study Major*** |  |  |
| Health & Medicine | 8 | 29.6 |
| Information Technology | 5 | 18.5 |
| Finance and Commerce | 5 | 18.5 |
| Business and Marketing | 4 | 14.8 |
| Psychology | 2 | 7.4 |
| Engineering | 1 | 3.7 |
| Architecture | 1 | 3.7 |
| Science | 1 | 3.7 |
| ***Country of origin*** |  |  |
| Afghanistan | 1 | 3.7 |
| Bangladesh | 2 | 7.4 |
| China | 3 | 11.1 |
| Colombia | 1 | 3.7 |
| Ghana | 1 | 3.7 |
| Hong Kong | 1 | 3.7 |
| India | 2 | 7.4 |
| Indonesia | 1 | 3.7 |
| Japan | 1 | 3.7 |
| Korea | 2 | 7.4 |
| Mexico | 1 | 3.7 |
| Mongolia | 1 | 3.7 |
| Nigeria | 2 | 7.4 |
| Philippines | 1 | 3.7 |
| Singapore | 1 | 3.7 |
| Taiwan | 1 | 3.7 |
| UK | 1 | 3.7 |
| Vietnam | 4 | 14.8 |
| ***Institution*** |  |  |
| University of Melbourne | 18 | 66.7 |
| RMIT University | 3 | 11.1 |
| Latrobe University | 3 | 11.1 |
| Monash University | 2 | 7.4 |
| Deakin University | 1 | 3.7 |
| ***Previously considered self-harm*** |  |  |
| No | 13 | 48.1 |
| Yes | 13 | 48.1 |
| Missing | 1 | 3.7 |
| ***Previously self-harmed*** |  |  |
| No | 19 | 70.4 |
| Yes | 7 | 25.9 |
| Missing | 1 | 3.7 |
| ***Previous suicidal ideation*** |  |  |
| No | 11 | 40.7 |
| Yes | 15 | 55.6 |
| Missing | 1 | 3.7 |
| ***Previous suicide attempt*** |  |  |
| No | 22 | 81.5 |
| Yes | 4 | 14.8 |
| Missing | 1 | 3.7 |
| ***Employment status*** |  |  |
| Full time student | 15 | 55.6 |
| Studying and working | 7 | 25.9 |
| Studying and looking for work | 4 | 14.8 |
| Missing | 1 | 3.7 |
| ***Highest attained education*** |  |  |
| Senior High School | 6 | 22.2 |
| Some University | 4 | 14.8 |
| Bachelor's degree | 10 | 37.0 |
| Postgraduate degree | 6 | 22.2 |
| Missing | 1 | 3.7 |

**Supplementary Table 2.** Proportion of participants endorsing potential workshop goals

| **Item** | **Students** | **Stakeholders** |
| --- | --- | --- |
| Opportunity for my voice to be heard | 33.3 | 23.8 |
| Be involved in improving self-harm and suicide prevention research | 74.1 | 81.0 |
| Opportunity to advocate for support for international students in my community | 85.2 | 66.7 |
| Better understand self-harm and suicide prevention research | 66.7 | 57.1 |
| Better understand how online tools could prevent suicide among international students | 66.7 | 76.2 |
| Work collaboratively with others | 59.3 | 76.2 |
| Connect socially with others | 40.7 | 23.8 |
| Gain understanding about how co-design can help improve self-harm and suicide prevention outcomes | 51.9 | 52.4 |
| Opportunities to connect with professional researchers and designers | 55.6 | 61.9 |
| I have no expectations for the co-design workshop | 3.7 | 4.8 |
| Other | 0 | 0.0 |

Note: Participants could select multiple options, so the totals do not add up to 100%. Student n = 26 (% missing = 3.7%), stakeholder n = 20 (% missing = 4.8%).

**Supplementary Table 3.** Detailed stakeholder demographic data

| **Item** | **Count** | **Proportion** |
| --- | --- | --- |
| ***Affiliation*** |  |  |
| University | 12 | 57.1 |
| Tafe | 5 | 23.8 |
| English language college | 1 | 4.8 |
| Housing provider | 1 | 4.8 |
| Law enforcement | 1 | 4.8 |
| Missing | 1 | 4.8 |
| ***Roles with international students*** |  |  |
| Administration | 3 | 14.3 |
| Housing | 4 | 19.0 |
| Teaching | 1 | 4.8 |
| Mental health services | 12 | 57.1 |
| Academic advising | 1 | 4.8 |
| Career services | 0 | 0.0 |
| Student affairs | 7 | 33.3 |
| Counselling services | 12 | 57.1 |
| Language support services | 2 | 9.5 |
| Financial aid and scholarships | 2 | 9.5 |
| Cultural and social activities coordination | 3 | 14.3 |
| Health services | 3 | 14.3 |
| Legal and immigration support | 1 | 4.8 |
| Orientation and transition programs | 7 | 33.3 |
| Student clubs and organisations | 1 | 4.8 |
| Tutoring and academic support services | 0 | 0.0 |
| Community engagement and outreach | 6 | 28.6 |
| IT and technology support | 0 | 0.0 |
| Other | 1 | 4.8 |
| No selection (all 0) | 1 | 4.8 |
| Missing | 1 | 4.8 |

Note: Participants could select multiple options for roles, so the totals do not add up to 100%. Stakeholder n = 20 (% missing = 4.8%).
